# Individualized Functional Parcellation of the Human Amygdala Using a Semi-supervised Clustering Method: A 7T Resting State fMRI Study

**DOI:** 10.3389/fnins.2018.00270

**Published:** 2018-04-26

**Authors:** Xianchang Zhang, Hewei Cheng, Zhentao Zuo, Ke Zhou, Fei Cong, Bo Wang, Yan Zhuo, Lin Chen, Rong Xue, Yong Fan

**Affiliations:** ^1^State Key Laboratory of Brain and Cognitive Science, Beijing MR Center for Brain Research, Institute of Biophysics, Chinese Academy of Sciences, Beijing, China; ^2^College of Life Sciences, University of Chinese Academy of Sciences, Beijing, China; ^3^Department of Biomedical Engineering, School of Bioinformatics, Chongqing University of Posts and Telecommunications, Chongqing, China; ^4^College of Psychology and Sociology, Shenzhen University, Shenzhen, China; ^5^Center for Language and Brain, Shenzhen Institute of Neuroscience, Shenzhen, China; ^6^Shenzhen Key Laboratory of Affective and Social Cognitive Science, Shenzhen University, Shenzhen, China; ^7^Beijing Institute for Brain Disorders, Beijing, China; ^8^Department of Radiology, Perelman School of Medicine, University of Pennsylvania, Philadelphia, PA, United States

**Keywords:** 7T, amygdala, parcellation, semi-supervised clustering, resting-state fMRI

## Abstract

The amygdala plays an important role in emotional functions and its dysfunction is considered to be associated with multiple psychiatric disorders in humans. Cytoarchitectonic mapping has demonstrated that the human amygdala complex comprises several subregions. However, it's difficult to delineate boundaries of these subregions *in vivo* even if using state of the art high resolution structural MRI. Previous attempts to parcellate this small structure using unsupervised clustering methods based on resting state fMRI data suffered from the low spatial resolution of typical fMRI data, and it remains challenging for the unsupervised methods to define subregions of the amygdala *in vivo*. In this study, we developed a novel brain parcellation method to segment the human amygdala into spatially contiguous subregions based on 7T high resolution fMRI data. The parcellation was implemented using a semi-supervised spectral clustering (SSC) algorithm at an individual subject level. Under guidance of prior information derived from the Julich cytoarchitectonic atlas, our method clustered voxels of the amygdala into subregions according to similarity measures of their functional signals. As a result, three distinct amygdala subregions can be obtained in each hemisphere for every individual subject. Compared with the cytoarchitectonic atlas, our method achieved better performance in terms of subregional functional homogeneity. Validation experiments have also demonstrated that the amygdala subregions obtained by our method have distinctive, lateralized functional connectivity (FC) patterns. Our study has demonstrated that the semi-supervised brain parcellation method is a powerful tool for exploring amygdala subregional functions.

## Introduction

The amygdala plays an important role in multiple emotional functions such as fear conditioning (Paton et al., 2006), emotion regulation (Blair et al., 2007), social behavior (Adolphs, 2010) and reward learning (Murray, 2007; Wassum and Izquierdo, 2015) both in animals and humans (LeDoux, 2007; Janak and Tye, 2015). Additionally, dysfunction of amygdala is considered to be associated with a variety of psychiatric disorders, such as depression, schizophrenia, autism, and anxiety (Phillips et al., 2003; Phelps and LeDoux, 2005).

Studies showed that the anatomic basis of multiple functions of amygdala is its complex internal structure (Kalin, 2004). Tract-tracing studies in animals have revealed that the amygdala complex consists of numerous nuclei, and each nucleus is uniquely connected with other brain areas (Morrison and Salzman, 2010). These nuclei can be broadly divided into a centromedial (CM) group, laterobasal (LB) group, and superficial (SF) group. The LB group is considered to bidirectionally connect with cortical areas, including the orbital prefrontal cortex, the hippocampus, as well as the sensory association areas, and play a vital role in updating current stimulus value associations. The CM group is thought to mediate behavioral responses to conditioned and unconditioned stimuli through its projection to hypothalamus, basal forebrain, and brainstem. The SF group has strong connections with olfactory cortex, insular, ventral striatum, and parahippocampal gyrus, which is consistent with its role in processing socially relevant information including olfactory and emotional stimuli (Pitkänen et al., 1997; Ghashghaei and Barbas, 2002; Sah et al., 2003; Kalin, 2004; Morrison and Salzman, 2010).

Amygdala nuclei's function has been extensively investigated in animal models using invasive methods. However, in humans, most of our understandings about amygdala function were obtained through postmortem examination or using noninvasive techniques, in which functional magnetic resonance imaging (fMRI) has been widely used. Nevertheless, researches that employed fMRI, especially resting-state fMRI (rs-fMRI), to explore functions of amygdala *in vivo* were mainly at the level of the whole region rather than its nuclei (LeDoux, 2007; Kim et al., 2011). One important reason for this is the absence of an effective approach to specify functionally distinct subregions of human amygdala. In fMRI studies, for activation or functional connectivity (FC) analysis, a prerequisite step is to define functionally distinct brain area as a region of interest (ROI). However, located deeply in the temporal lobe and small in size, the amygdala is difficult to be parcellated into meaningful subregions in the living human brain, even if using the high-resolution structural MRI (Saygin et al., 2017). Thus, the ability to anatomically and functionally characterize these subregions is of great significance to understand the amygdala physiology and help diagnose diseases involving the amygdala.

Different strategies have been proposed to identify the human amygdala subregions, including: (i) using probabilistic atlases constructed based on cytoarchitectonic boundaries (Amunts et al., 2005) or based on *ex vivo* high-resolution MRI scanning of autopsy brains (Saygin et al., 2017); (ii) application of clustering methods to *in vivo* neuroimaging data. Among the probabilistic atlases, the Julich cytoarchitectonic atlas (Eickhoff et al., 2005) is considered to be a histologically accurate definition of amygdala subregions and has been widely used in fMRI studies (Ball et al., 2007; Roy et al., 2009; Li et al., 2012; Gabard-Durnam et al., 2014; Qin et al., 2014; Engman et al., 2016; Rausch et al., 2016; Eckstein et al., 2017). However, while the regions defined by the atlas are cytoarchitectonicly homogeneous, they do not necessarily have homogeneous functional signals or connectivity. Their rationality for resting state FC analysis has yet to be determined (Craddock et al., 2012). The other strategy, brain parcellation based on neuroimaging data, could be modeled as a data clustering problem to group brain image voxels into clusters, and a variety of clustering algorithms have been adopted to parcellate the amygdala. Most existing studies adopted unsupervised clustering algorithms, such as spectral clustering (Solano-Castiella et al., 2010, 2011; Bzdok et al., 2013), k-means (Bach et al., 2011), and self-organizing map (Mishra et al., 2014) to parcellate the amygdala based on voxel similarity derived from the neuroimaging data. Particularly, Mishra et al. (2014) and Bickart et al. (2012) have demonstrated the feasibility of parcellating human amygdala using resting state fMRI data. However, the unsupervised clustering typically identified amygdala subregions at a group level and these methods cannot be applied to individual subject to obtain subregions consistent across subjects (Craddock et al., 2012; Mishra et al., 2014). Furthermore, these amygdala parcellation results were typically derived from 3T fMRI data with relatively low spatial resolution, which might largely reduce the spatial specificity of the parcellation results.

Recently, a semi-supervised spectral clustering (SSC) based brain parcellation method has been proposed to parcellate brain area based on resting-state fMRI data (Kulis et al., 2009; Cheng and Fan, 2014). Compared with the unsupervised clustering techniques, the semi-supervised brain parcellation method possesses a remarkable superiority that it can integrate prior information, derived from the existing neuroanatomy knowledge (such as cytoarchitectonic mapping results or meta-analysis results) to guide the clustering. In this study, we extend the SSC approach by combining it with the automatic extraction of whole human amygdala using FreeSurfer (Fischl et al., 2002) to parcellate human amygdala into functional subregions at an individual subject level. Besides, to guarantee the spatial specificity of parcellation results, a high resolution 7 Tesla (7T) rs-fMRI dataset was used in this study.

We hypothesized that the proposed method could parcellate human amygdala into several functionally homogeneous and spatially coherent subregions based on the high resolution 7T rs-fMRI data at an individual level. We designed to evaluate our method in terms of its capability to capture the inter-subject variability at first. Then, the functional homogeneity of the obtained subregions were assessed by a comparison with the cytoarchitectonic atlas. At last, amygdala subregional FC patterns would be investigated with respect to their distinctiveness and asymmetry between hemispheres. Our aim was to achieve an accurate and individualized functional parcellation of amygdala and provide a reliable alternative for rs-fMRI studies that investigated subregional function of amygdala or other brain areas.

## Materials and methods

### Participants

Twenty healthy Chinese-speaking subjects (mean age 23.6 ± 3.5 years, 8 males) with no history of mental disorders, neurological disorders, or intracranial surgeries participated in the study, who were recruited from local universities between October 2015 and January 2016. Exclusion criteria include following aspects: (1) self-reported sleep during the procedure of MRI scanning; (2) head motion criteria of mean frame-wise displacement (FD) >0.2 mm (Power et al., 2014).

This study was carried out in accordance with the recommendations of the institutional review board of Beijing MRI Center for Brain Research with written informed consent from all subjects. All subjects gave written informed consent in accordance with the Declaration of Helsinki. The protocol was approved by the institutional review board of Beijing MRI Center for Brain Research.

### Data acquisition

Both rs-fMRI and structural MRI (sMRI) data were acquired from each subject at 7T. For rs-fMRI, subjects were instructed to lie supine on the scanner bed with eyes closed, not thinking anything in particular and staying awake during scanning. The rs-fMRI data were acquired using a Siemens 7T Magnetom scanner (Erlangen, Germany) equipped with a Nova Medical 32-channel head coil. A single-shot, gradient recalled echo planar imaging (GRE-EPI) sequence was used to collect the 7T rs-fMRI data with the following parameters: time repetition [TR] = 3,000 ms, time echo [TE] = 22 ms, flip angle [FA] = 70°, number of axial slices = 70, field of view [FOV] = 192 × 192 mm^2^, voxel size = 1.5 × 1.5 × 1.5 mm^3^, accelerating factor (integrated parallel acquisition technique [iPAT]) = 3, number of measurements (or volumes) = 200, acquisition time[TA] = 10:23 min. The high-resolution 3D T1-weighted (T1w) anatomical data at 7T were acquired using a magnetization-prepared rapid acquisition gradient echo (MPRAGE) sequence in sagittal direction with the following parameters: TR = 2,200 ms, TE = 3.21 ms, inversion time [TI] = 1,100 ms, FA = 7°, FOV = 224 × 224 mm^2^, voxel size = 0.7 × 0.7 × 0.7 mm^3^.

The extra sMRI data was acquired for each subject using a Siemens 3T Prisma MR scanner (Erlangen, Germany) equipped with a Siemens 20-channel head coil. The sMRI was acquired in sagittal orientation using a MPRAGE sequence with the following parameters: TR = 2,530 ms, TE = 3.37 ms, TI = 1,100 ms, FOV = 256 × 256 mm^2^, FA = 7°, iPAT = 2, voxel size = 1.0 × 1.0 × 1.0 mm^3^.

During the data acquisition, foam pads were used to restrain head motion, and scanner noise was shielded with earplugs.

### Data preprocessing

The rs-fMRI data was preprocessed using SPM12 (http://www.fil.ion.ucl.ac.uk/spm) and Data Processing and Analysis of Brain Imaging (DPABI v2.2) (Yan et al., 2016). Prior to preprocessing, the first 10 volumes of the rs-fMRI data from each subject were discarded to allow the signal to reach equilibrium. The preprocessing included following steps. Firstly, the remaining 190 volumes were corrected for acquisition time delay between different slices. Secondly, each volume of the rs-fMRI data after slice timing was realigned to the mean image of all the volumes to correct for the head motion. Thirdly, the individual 7T sMRI data was co-registered to the same subject's mean functional image after motion correction. Next, the 7T sMRI was further co-registered to this subject's 3T sMRI, and the motion corrected rs-fMRI data was subsequently co-registered to the 3T sMRI by applying the linear transformation of the co-registration between the 7T sMRI and 3T sMRI for the same subject. Consequently, the co-registered rs-fMRI data can be nonlinearly normalized to MNI space with the deformation field calculated by the DARTEL tool based on the unified segmentation of the 3T sMRI. Then, linear and quadratic trends were removed from the co-registered rs-fMRI data, and temporal band-pass filtering (0.01–0.08 Hz) was further performed to reduce the low-frequency drift and high-frequency noise. Moreover, the nuisance variables, including averaged cerebrospinal fluid (CSF) signal, averaged white matter (WM) signal, global mean signal, and 24 motion parameters (Friston et al., 1996), were regressed out to reduce the physiological and motion artifacts. Finally, the regressed rs-fMRI data was nonlinearly normalized to MNI space with a spatial resolution of 1.5 × 1.5 × 1.5 mm^3^ using the transformation parameters produced by DARTEL tool, and then was spatially smoothed with a 4 mm full width at half maximum Gaussian kernel.

Regional homogeneity (ReHo) (Zang et al., 2004), which measured each voxel's functional consistency with its neighboring voxels, was computed before the spatially smoothing. The ReHo was calculated to obtain the prior information for functional parcellation of the amygdala.

### Individualized functional parcellation of the amygdala

**Figure 1:**
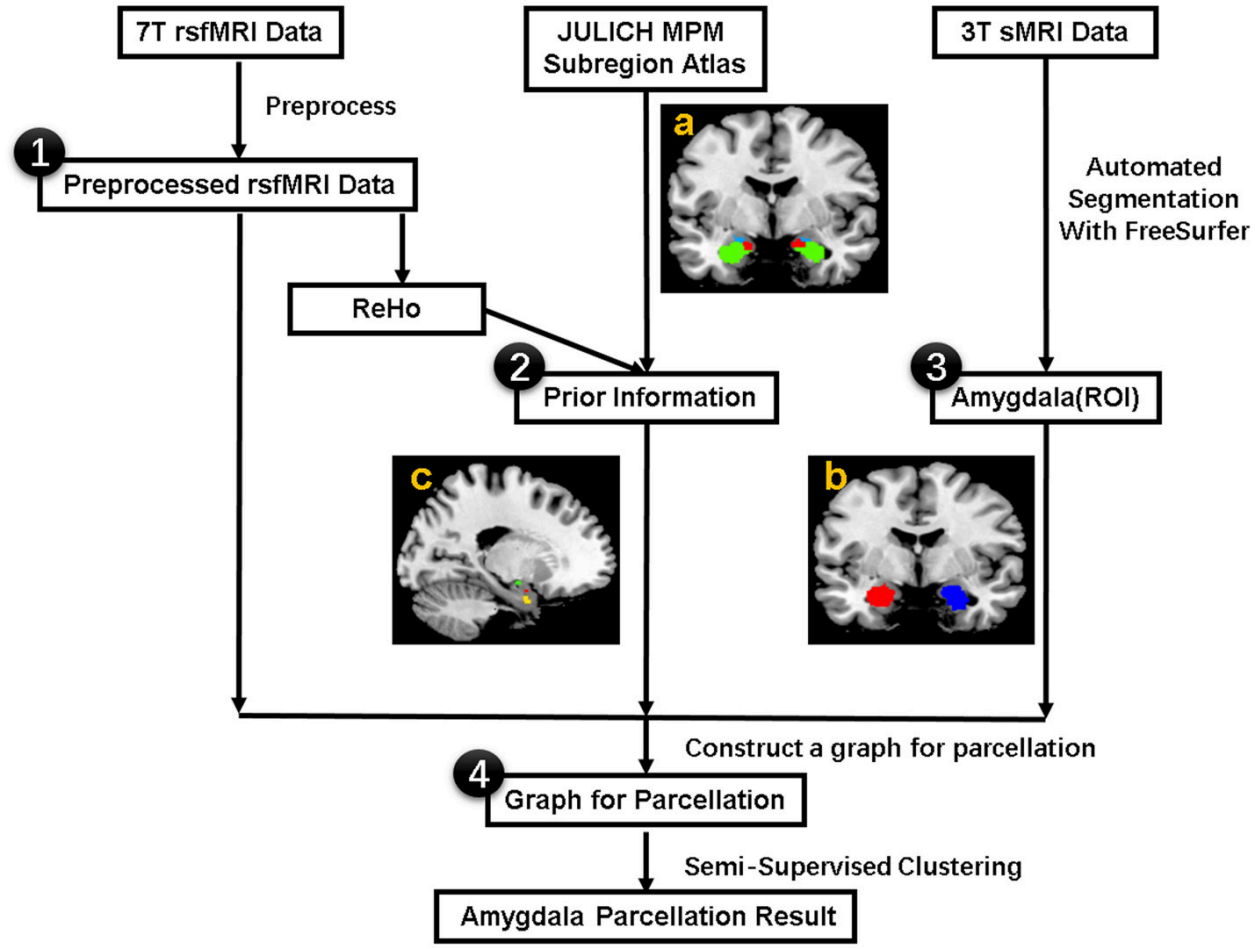
The procedure for the individualized functional parcellation of the amygdala with semi-supervised spectral clustering algorithm based on the 7T rs-fMRI data. **(a)** Julich atlas from the SPM Anatomy toolbox. **(b)** Bilateral amygdala masks. **(c)** Three small regions derived from the Julich atlas, which will be used as prior information in the parcellation. The procedure consists of four basic steps: (1) Preprocess 7T rsfMRI data, (2) Acquire prior information from Julich atlas based on the ReHo data, (3) Extract bilateral amygdala masks by segmenting individual 3T sMRI data with FreeSurfer, (4) Transfer the amygdala segmentation problem to graph partition issue by modeling each voxel in amygdala as graph node and connecting each pair of nodes with an edge weighted by the similarity of fMRI signal, finally segment the graph with a semi-supervised clustering algorithm. rsfMRI = resting state fMRI, ReHo = Regional Homogeneity, ROI = Region of Interest. sMRI = structural MRI.

The flowchart for individualized functional parcellation of amygdala is shown in Figure 1, consisting of amygdala extraction, similarity graph construction and amygdala parcellation using the SSC algorithm (Zhang et al., 2017).

#### Extraction of the amygdala

For each subject, the bilateral amygdalae were extracted based on the 3T sMRI using the FreeSurfer v5.3.0 (Fischl et al., 2002). The extracted bilateral amygdalae were further normalized to the MNI space with the deformation field produced by the SPM's DARTEL tool with the unified segmentation of the individual 3T sMRI.

#### Individualized parcellation of the amygdala based on the functional signals

##### Framework for the SSC based amygdala parcellation

The semi-supervised spectral clustering algorithm (Kulis et al., 2009; Cheng and Fan, 2014) was adopted to parcellate the amygdala based on the preprocessed 7T rs-fMRI data in the study. The SSC based amygdala parcellation has following two main steps.

The SSC algorithm firstly constructs a graph G = (V, E) by modeling all the voxels within the amygdala as graph nodes V and connecting each pair of amygdala voxels with an edge E weighted by the nonnegative similarity of their functional signals. Consequently, the similarity is defined as Craddock et al. (2012):

(1) $$\begin{array}{l} f_{uv}=r_{uv}+1, \end{array}$$

where *r*_*uv*_ ∈ [−1, 1] is the FC between amygdala voxels *u* and *v*, measured by Pearson correlation coefficient of functional signals. Actually, the constructed graph G can be represented as a *NxN* similarity matrix with elements composed by edge weights *f*_*uv*_, and N is the number of voxels in the amygdala considered for parcellation.

Then, the constructed graph G is partitioned into *n* subgraphs (*n* = 3 amygdala subregions in the present study). The graph partition problem is solved through optimization of (i) the similarity of nodes within each subgraph and (ii) the similarity between the parcellation result and prior information.

In the SSC based amygdala parcellation, the prior information is obtained based on the maximum probability map (MPM) of the amygdala cytoarchitectonic parcellation with three subregions (CM, LB, and SF) from the Julich atlas (Eickhoff et al., 2005). To avoid possible bias to the anatomical priors that might be inconsistent with the functional information, instead of directly using the Julich atlas, we selected three small regions with most homogeneous functional signals from the Julich MPM as prior information with the following procedures. Firstly, the amygdala cytoarchitectonic subregions are segmented into a number of small functionally homogeneous regions according to each voxel's functional consistency with its neighboring voxels (ReHo) using the watershed segmentation algorithm. Then, three homogeneous regions, *p*_*i*_, *i* = 1, …3, one from each amygdala cytoarchitectonic subregion, with the smallest MinMaxCut (Mcut) value, as defined by Equation (2), are selected as prior information. The Mcut value is computed as (Ding et al., 2001):

(2) $$\begin{array}{l} M_{cut}=\sum_{i\;=\;1}^{n}\frac{\sum_{j\;=\;1,j\;\neq \;i}^{n}{\;\sum }_{u\in p_{i},v\in p_{j}\;}f_{uv}}{\sum_{u,v\in p_{i},u\;\neq \;v\;}f_{uv}}, \end{array}$$

where *n* = 3 is the number of subgraphs, *p*_*i*_ is the small region which will be selected as prior information, *f*_*uv*_ is the similarity measure defined in Equation (1).

##### Implementation of the semi-supervised spectral clustering

Under the above described framework for amygdala parcellation, the SSC algorithm is implemented by formulating two terms, namely the data term and the prior term. Among them, the data term defines the similarity of nodes within subgraphs, and the prior term defines the similarity between the parcellation result and the prior information.

Specially, the normalized cut (Shi and Malik, 2000; Craddock et al., 2012) is adopted as data term to measure the similarity of nodes within subgraphs:

(3) $$\begin{array}{l} \sum_{c=1}^{n}\frac{\sum_{u,v\in g_{c},u\neq v\;}f_{uv}}{\sum_{u\in g_{c},v\in G}f_{uv}}, \end{array}$$

where *n* is the number of subgraphs, *g*_*c*_, *c* = 1, …, *n* represents each subgraph of the graph G, and *f*_*uv*_ is the similarity measure defined in Equation (1).

At the same time, the prior term, which defines the similarity between the parcellation result and the prior information, is formulated by:

(4) $$\begin{array}{l} \sum_{c\;=\;1}^{n}\frac{\sum_{i\;=\;1}^{n}\sum_{u,v\in p_{i}\cap g_{c}\;}f_{uv}}{\sum_{u\in g_{c},v\in G}f_{uv}}, \end{array}$$

where *n* is the number of subgraphs, and *p*_*i*_ = 1, …, *n* is the small region with homogeneous functional signals that is adopted as the prior information, which is derived from the cytoarchitectonic MPM.

Then, the graph partition issue is transferred to the optimization of the following objective function:

(5) $$\begin{array}{l} \text{J}\left({\left\{g_{c}\right\}}_{c\;=\;1}^{n}\right)=argmax\sum_{c\;=\;1}^{n}\frac{\sum_{u,v\in g_{c},u\neq v\;}f_{uv}}{\sum_{u\in g_{c},v\in G}f_{uv}}\\ \;+\;\lambda (\alpha \sum_{c\;=\;1}^{n}\frac{\sum_{i\;=\;1}^{n}\sum_{u,v\in p_{i}\cap g_{c}\;}f_{uv}}{\sum_{u\in g_{c},v\in G}f_{uv}}\\ \;+\;(1-\alpha )\sum_{c\;=\;1}^{n}\frac{\sum_{u,v\in g_{c}}e_{uv}}{\sum_{u\in g_{c},v\in G}f_{uv}}), \end{array}$$

where *e*_*uv*_ is equal to *f*_*uv*_ if voxels *u* and *v* are spatially nearest neighbors, or 0 otherwise. λ and α ∈ [0, 1] are weighting factors, and are set to 2 and 0.5 in this work, respectively.

Finally, the optimization problem of the objective function defined in Equation (5) is solved by an improved weighted kernel k-means algorithm (Cheng and Fan, 2014).

### Inter-subject variability of the individualized functional parcellation results

To evaluate the inter-subject variability of the individualized functional parcellation results, volume measurements and probability map analyses were conducted, which was similar to the way carried out by Amunts et al (Amunts et al., 2005).

Firstly, volumes of the bilateral amygdala and their parcellated three subregions were calculated for each of the 20 subjects. The volume is defined as:

(6) $$\begin{array}{l} ROI\;Volume=Voxel\;Numbers\text{ X }Voxel\;Size, \end{array}$$

where *ROI* represents bilateral amygdalae and each of their subregions, and *Voxel Size* is 1.5 × 1.5 × 1.5 mm^3^.

Then, the probability maps and MPM were computed. The probability maps describe, for each voxel that has a label in anyone of the subjects, frequency with which a SSC parcellation subregion (or label) is observed at this specific voxel in the sample of 20 subjects. Based on the calculated probability maps, the MPM (denoted as SSC-MPM), which describes the most probable SSC parcellation subregion at each voxel, is computed according to the following rules: (i) For each voxel, the accumulative probability (or frequency) of this voxel across three SSC subregions is higher than 60%, or the probability for any one of the three subregions is higher than 50%; otherwise, the voxel will not be assigned with label. (ii) The voxel will be assigned to that subregion, which shows the highest probability at this specific position. (iii) If a voxel has the same probability for the three subregions, it will be assigned to that subregion which has highest average probability across its 3 × 3 × 3 neighbors. It should be noted that the threshold 60% for including a voxel into the MPM is determined based on the relationship between the whole amygdala volume of SSC-MPM and the number of subjects who have a label (that is, share the same voxel) at this specific voxel (see **Figure 4** in Results section). The whole amygdala volume of SSC-MPM is around 2 cm^3^ if the threshold is set to 60% (that is, SSC subregions were found at this voxel in at least 12 of the 20 subjects.), which is consistent with the mean volume of whole amygdala estimated according to the volume measurements (see Table 1). The resulting MPM generates a contiguous and nonoverlapping parcellation of amygdala subregions.

**Table 1:** Volume (Mean ± SE, mm^3^, *N* = 20) of the whole amygdala and each amygdala subregion: centromedial (CM), laterobasal (LB), superficial (SF) amygdala.

**Amygdala structure**	**Left**	**Right**
CM	420.69 ± 61.11	276.58 ± 51.43
LB	1,391.68 ± 86.53	1,442.48 ± 69.42
SF	229.33 ± 38.10	317.42 ± 34.48
Whole	2,041.71 ± 33.46	2,036.48 ± 45.24

### Assessment of the similarity between the functional and cytoarchitectonic parcellations

In order to evaluate the correspondence between our functional parcellation results and cytoarchitectonic mapping of human amygdala, we used the Dice coefficient to assess the overlap degree between the subregions obtained by the proposed method and the cytoachitectonic subregions in each subject (Dice, 1945):

(7) $$\begin{array}{l} \text{Dice Coefficient}=\;\frac{2\left(X\cap Y\right)}{X+Y} \end{array}$$

The Dice's coefficient is the ratio of twice the number of voxels that have the same labels in both partitions, divided by the total number of voxels present in both partitions. Dice's coefficient results in numbers between zero and one. The larger number indicates better correspondence between partitions.

### Evaluation of the amygdala parcellation results based on the functional homogeneity

Functional homogeneity of the amygdala parcellation results was evaluated by a modified silhouette width (SI) measure that has been widely used to quantitatively assess the functional homogeneity of parcellation results (Craddock et al., 2012; Cheng et al., 2014; Mishra et al., 2014). Particularly, the modified SI is defined as:

(8) $$\begin{array}{l} SI_{n}=\frac{1}{n}\sum_{c=1}^{n}\frac{a_{c}-b_{c}}{\max\left\{a_{c},b_{c}\right\}}, \end{array}$$

where *SI*_*n*_ is the modified SI with *n* clusters, $a_{c}=\frac{1}{n_{c}\left(n_{c}-1\right)}\underset{u,v\in g_{c},u\neq v}{\sum }f_{uv}$ measures the intrinsic functional similarity between each pair of voxels *u* and *v* assigned to the cluster *g*_*c*_, $b_{c}=\frac{1}{n_{c}\left(N-n_{c}\right)}\underset{u\in g_{c},v\in \left(G-g_{c}\right)}{\sum }f_{uv}$ measures the similarity between an intra-cluster voxel *u* and an extra-cluster voxel *v*, *n*_*c*_ is the number of voxels assigned to the cluster *g*_*c*_, and *N* is the total number of voxels within the target region considered for parcellation.

For each subject, the modified SI was calculated for the SSC based functional parcellation and the cytoarchitectonic parcellation, respectively. Paired *t*-tests were performed to compare SI values of these two parcellation methods across all the subjects in this study.

### Comparison with a state of the art unsupervised clustering method

The semi-supervised clustering method was compared with a state of the art unsupervised brain parcellation method—normalized cut (NCUT) (Craddock et al., 2012). To be comparable with the SSC partition, the NCUT algorithm was applied to parcellate amygdala into 3 subregions bilaterally based on the similarity measure defined by Equation (1) for each of the 20 subjects. Each subregion of the parcellation results obtained by the NCUT algorithm was labeled according to its overlap with the cytoarchitectonic subregion. Namely, each subregion shares the same label with the cytoarchitectonic subregion if they have the largest overlapping area.

In the comparison experiments, the cross subject consistency of partitioning was used to assess the performance of the brain parcellation methods (Shen et al., 2010). The cross subject consistency can be measured using the average discrete entropy across all voxels in the target region considered for parcellation (Shen et al., 2010), which is defined as:

(9) $$\begin{array}{l} H=\;\frac{1}{N}\sum_{u}H\left(u\right), \end{array}$$

where *H* is the average discrete entropy, *N* is the total number of voxels within the target region, and *H*(*u*) is the discrete entropy at voxel *u*. The discrete entropy is used to assess uncertainty of label assignment at a single voxel *u*, which is defined as:

(10) $$\begin{array}{l} H\left(u\right)=-\sum_{k=1}^{3}Pr\left(u=k\right)\text{log}\left(Pr\left(u=k\right)\right), \end{array}$$

where *Pr*(*u* = *k*) is the probability of voxel *u* being classified as subregion *k*, and *k* = 1,2,3 is the label of the amygdala subregion. *Pr*(*u* = *k*) can be obtained from the amygdala subregion probability map.

The smaller the average entropy *H*, the better the consistency between partitions across all subjects. The average entropy of the SSC and NCUT parcellation results were calculated for left and right amygdala, respectively. In addition, the overlap between parcellation results obtained by the NCUT method and the cytoarchitectonic map was also calculated based on Equation (7).

### Functional connectivity analyses of the amygdala parcellation results

Firstly, FC patterns of the amygdala subregions were computed and their distinction was investigated. Furthermore, difference in FC patterns between the ipsilateral amygdala subregions was explored, and the asymmetry in FC patterns of bilateral amygdala subregions was examined as well.

#### Individual subject-level functional connectivity analysis

For each subject, each of the three amygdala subregions in each hemisphere was selected as the seed ROI. The individual-level FC maps were generated for each selected ROI by computing Pearson correlations between the mean time course extracted from the ROI and the time courses from the whole brain. The Pearson coefficients of resulting whole brain voxel-wise FC maps were converted to z values using the Fisher's r-to-z transformation for subsequent group-level statistical analysis.

#### Group-level statistical analysis

In the first place, one sample *t*-tests were applied to the individual-level FC maps of all the subjects for each ROI of the amygdala subregions to explore group-level FC patterns. Secondly, paired *t*-tests were applied to the individual-level FC maps of all the subjects between each pair of the ipsilateral amygdala's subregions in each hemisphere for exploring the difference in FC patterns. Furthermore, in order to examine the asymmetry in FC patterns, paired *t*-tests were applied to the individual-level FC maps of all the subjects between bilateral ROIs of the same amygdala subregion from two hemispheres. For above *t*-tests, only clusters, which were significant at a threshold of *p* < 0.001 and an extent threshold of *p* < 0.05 with cluster-level family-wise error correction, were reported.

## Results

### Individualized functional parcellation results of the amygdala

**Figure 2:**
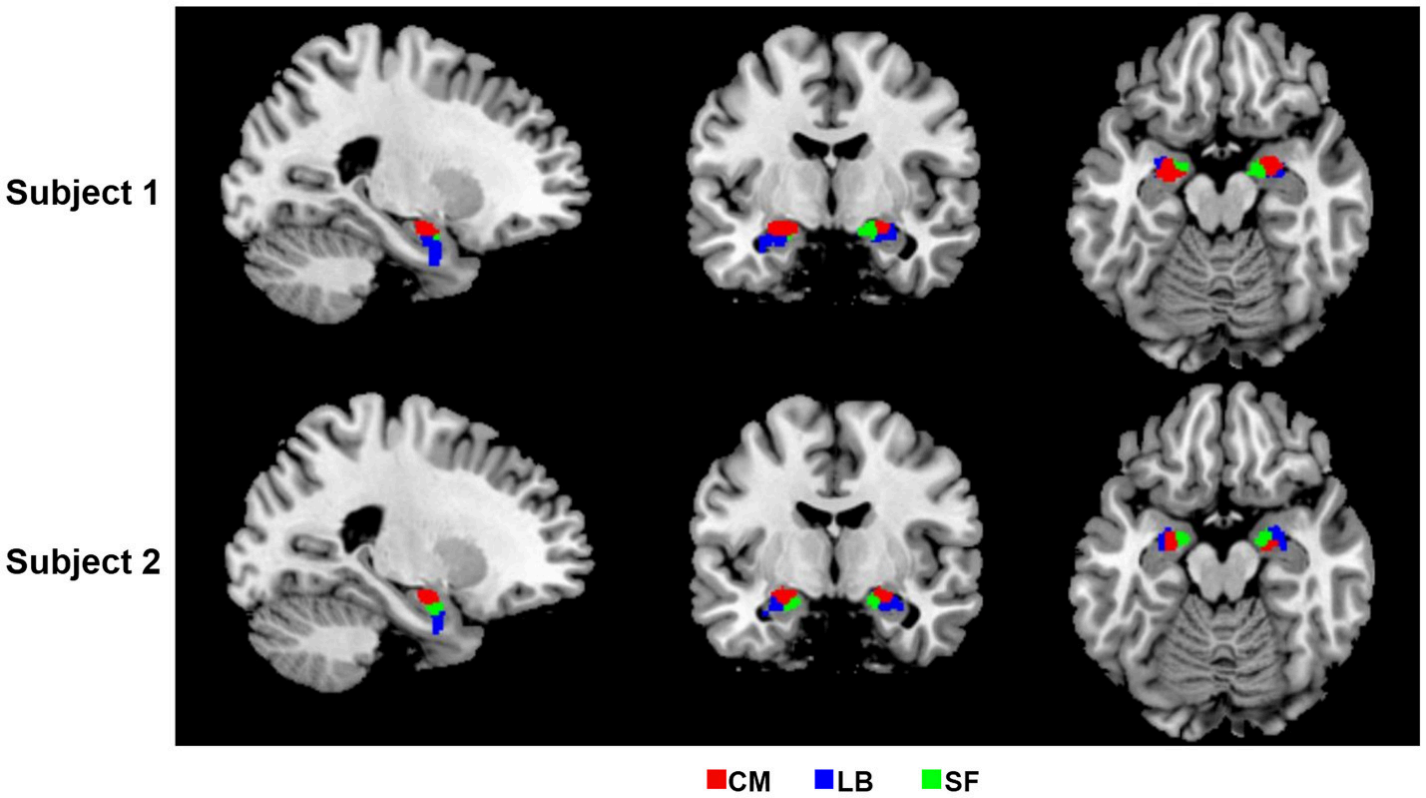
2D view of the obtained amygdala subregions in two randomly selected subjects, located at (−23, −7, −17). The results are overlaid on the standard template in the MNI space. CM, centromedial amygdala; LB, laterobasal amygdala; SF, superficial amygdala.

In the present study, bilateral amygdalae were successfully parcellated into three spatially coherent subregions for each subject, namely CM, LB, and SF, which were named after respective prior information. Parcellation results of two randomly selected subjects are shown in Figure 2.

### Inter-subject variability of the individualized functional parcellation results

#### Volume measurements

Table 1 shows the mean volumes of bilateral amygdalae and their parcellated three subregions for the 20 subjects. The mean volume of the whole amygdala is 2041.71 ± 33.46 mm^3^ (Mean ± SE) for left hemisphere, and is 2036.48 ± 45.24 mm^3^ (Mean ± SE) for right hemisphere.

#### Probability maps for the functional parcellation results of the amygdala

**Figure 3:**
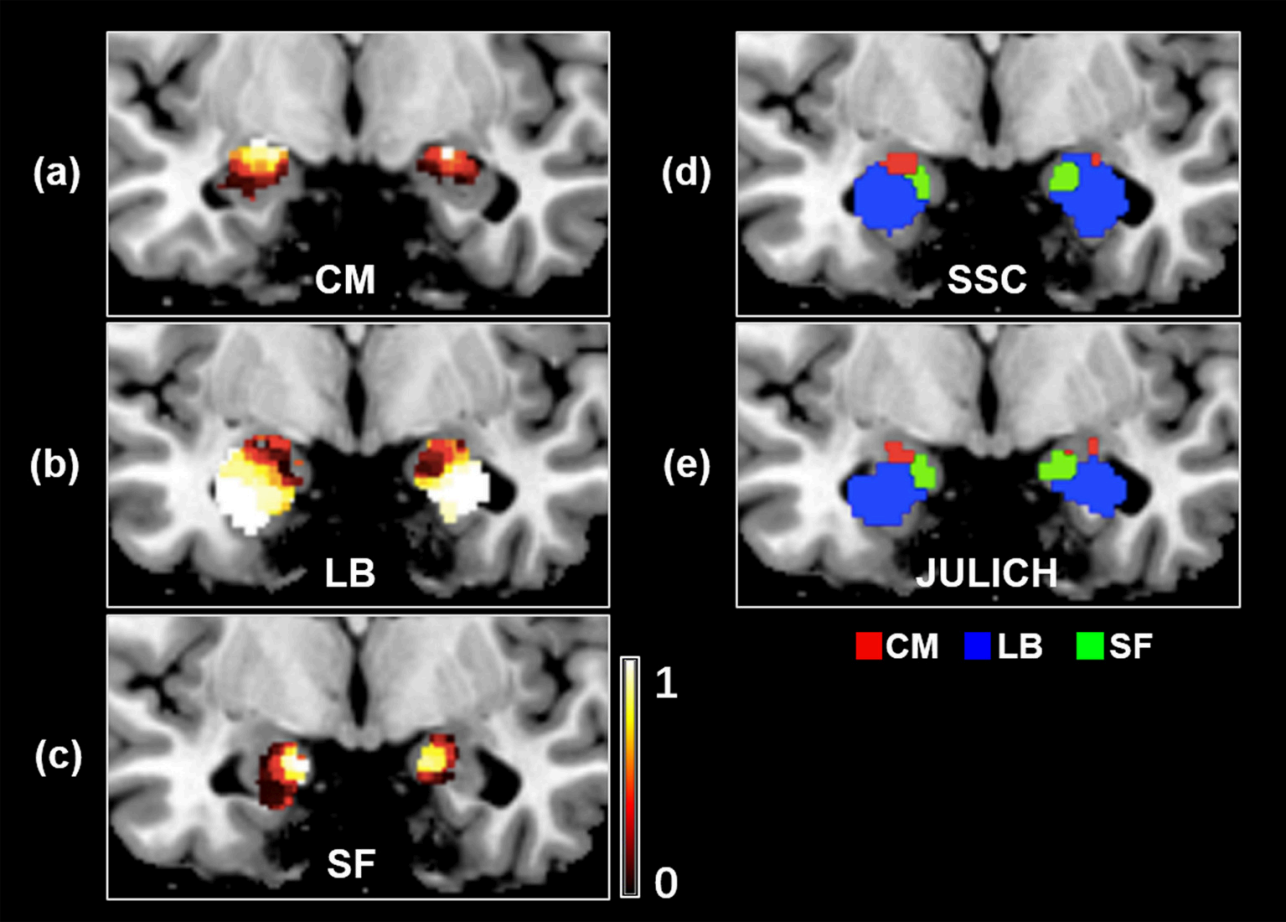
The probability map of amygdala's subregions obtained by the SSC-based brain parcellation method. **(a)** The probability map of the CM subregion, located at (−26, −7, −17) in the MNI space **(b)** the probability map of the LB subregion, located at (−26, −4, −17) in the MNI space **(c)** the probability map of the SF subregion, located at (−26, −5, −17) in the MNI space **(d)** the maximum probability map of the SSC-based functional parcellation **(e)** the maximum probability map of the Julich cytoarchitectonic atlas obtained from the SPM Anatomy toolbox. **(d,e)** views are located at (−19, −5, −19) in the MNI space. SSC, semi-supervised clustering; CM, centromedial; LB, laterobasal; SF, superficial.

**Figure 4:**
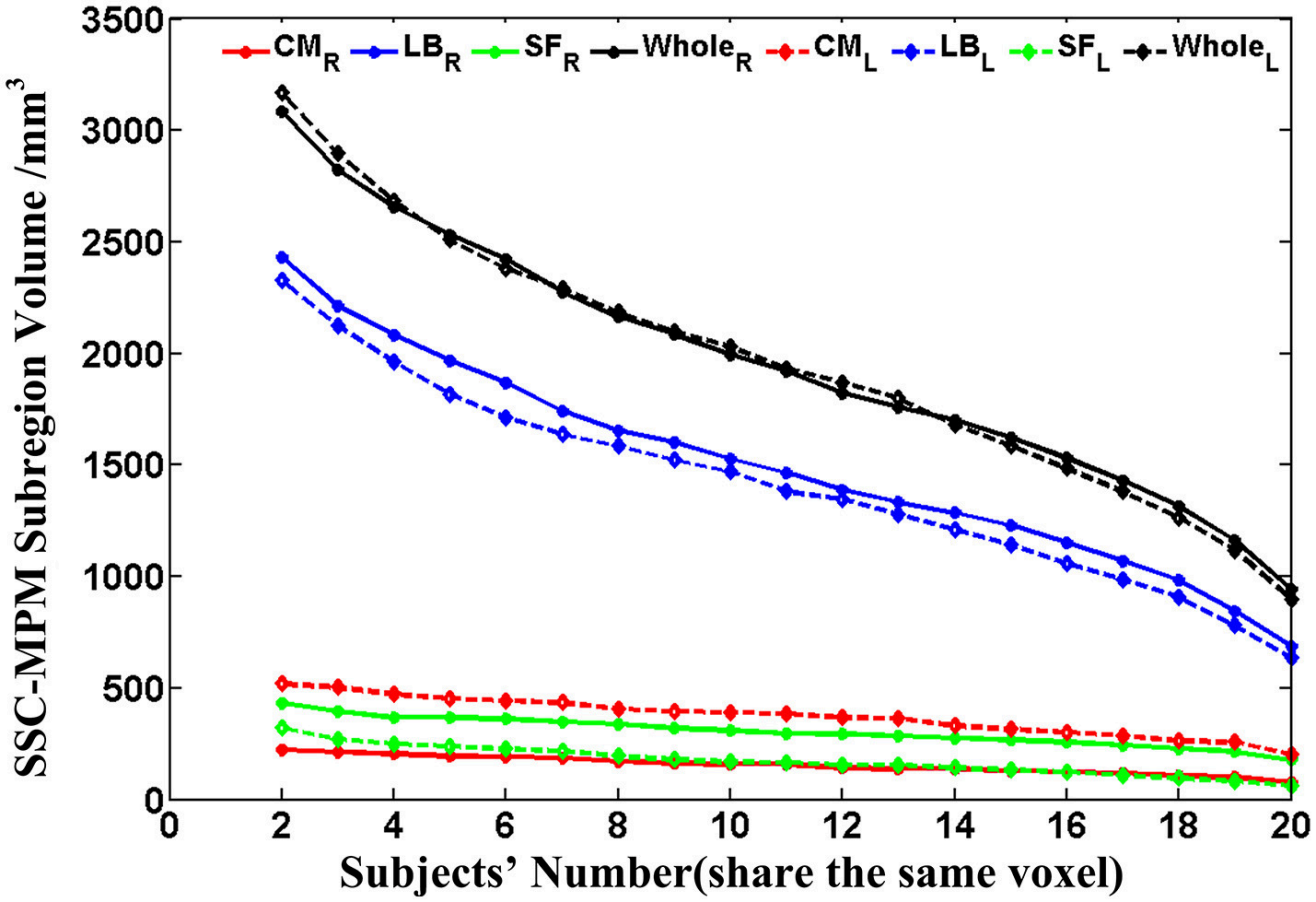
The volume variation of the whole amygdala and each subregion within the SSC-MPM with the change of the subjects' number that share the same voxel. The dotted and solid lines represent the left and right hemispheres, respectively. The volume of the whole amygdala and the CM, LB, as well as the SF subregion in the SSC-MPM was shown separately. SSC-MPM, semi-supervised clustering parcellation maximum probability map; CM, centromedial; LB, laterobasal; SF, superficial.

Probability maps for bilateral amygdala subregions, namely CM, LB, and SF, are shown in Figures 3a–c. Meanwhile, the obtained SSC maximum probability map (SSC-MPM) is shown in Figure 3d. For comparison, the cytoarchitectonic MPM is shown in Figure 3e as well. Besides, the relationship between the whole amygdala volume of SSC-MPM and the number of subjects who have a label (that is, share the same label) at one specific voxel is shown in Figure 4.

### Similarity between the functional and cytoarchitectonic parcellations

Table 2 shows the overlap degree between our functional parcellation results and the cytoarchitectonic map. The high mean Dice coefficients indicate our functionally parcellated subregions have a fine correspondence with the cytoarchitectonic subregions.

**Table 2:** Overlap between the SSC/NCUT parcellation and cytoarchitectonic parcellation. The overlap degree was measured using Dice Coefficients (Mean ± SE, *N* = 20).

		**CM**	**LB**	**SF**
NCUT	Left	0.490 ± 0.035	0.602 ± 0.018	0.224 ± 0.028
	Right	0.122 ± 0.029	0.450 ± 0.035	0.208 ± 0.052
SSC	Left	0.645 ± 0.031	0.781 ± 0.015	0.739 ± 0.041
	Right	0.547 ± 0.046	0.764 ± 0.018	0.756 ± 0.037

*NCUT, normalized cut; SSC, semi-supervised clustering; CM, centromedial amygdala; LB, laterobasal amygdala; SF, superficial amygdala; Left, left amygdala; Right, right amygdala*.

### Functional homogeneity of the parcellation results

**Figure 5:**
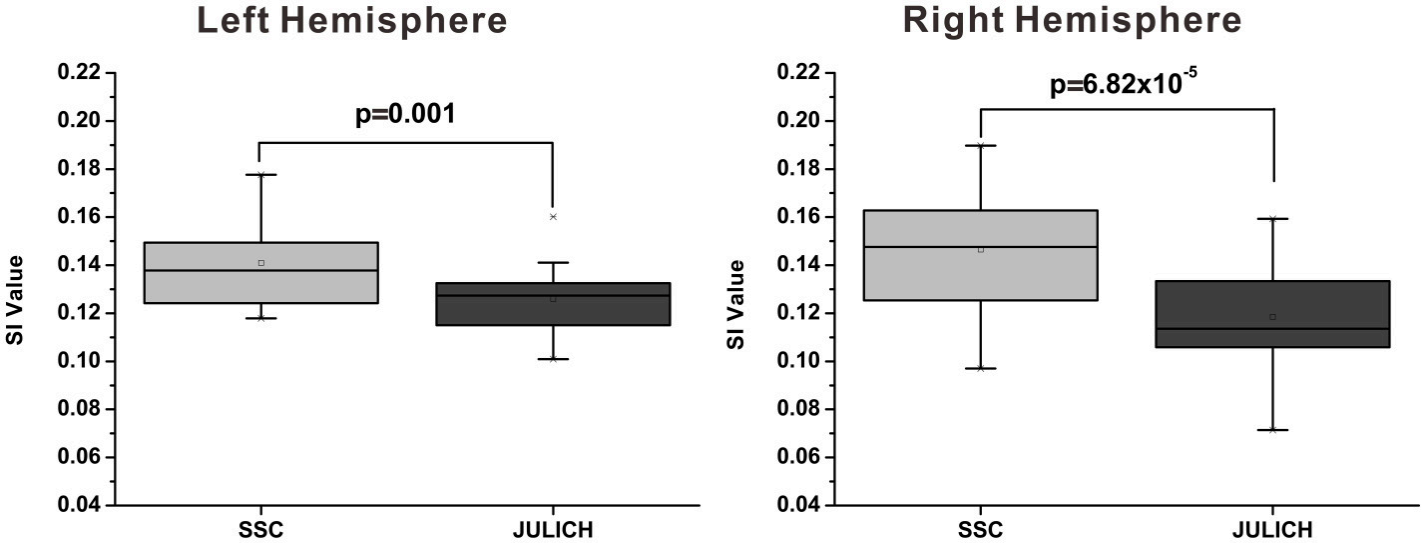
Functional homogeneity comparison between the SSC-based amygdala parcellation and the Julich cytoarchitectonic atlas. The functional homogeneity is measured by the modified SI index. SSC, semi-supervised clustering; SI, silhouette width.

The mean modified SI value of SSC based brain parcellation method is 0.141 ± 0.004 (Mean ± SE) for left hemisphere, and is 0.147 ± 0.006 (Mean ± SE) for right hemisphere; whereas the mean modified SI value of the cytoarchitectonic parcellation method is 0.126 ± 0.003 (Mean ± SE) for left hemisphere, and is 0.119 ± 0.005 (Mean ± SE) for right hemisphere. Results of paired *t*-tests have demonstrated that the modified SI values of the SSC based brain parcellation method are significantly larger than the cytoarchitectonic parcellation method with *p* = 0.001 and *p* = 6.82 × 10^−5^ for the left and right amygdala, respectively (Figure 5).

### Comparison with the state-of-the-art unsupervised parcellation method

**Figure 6:**
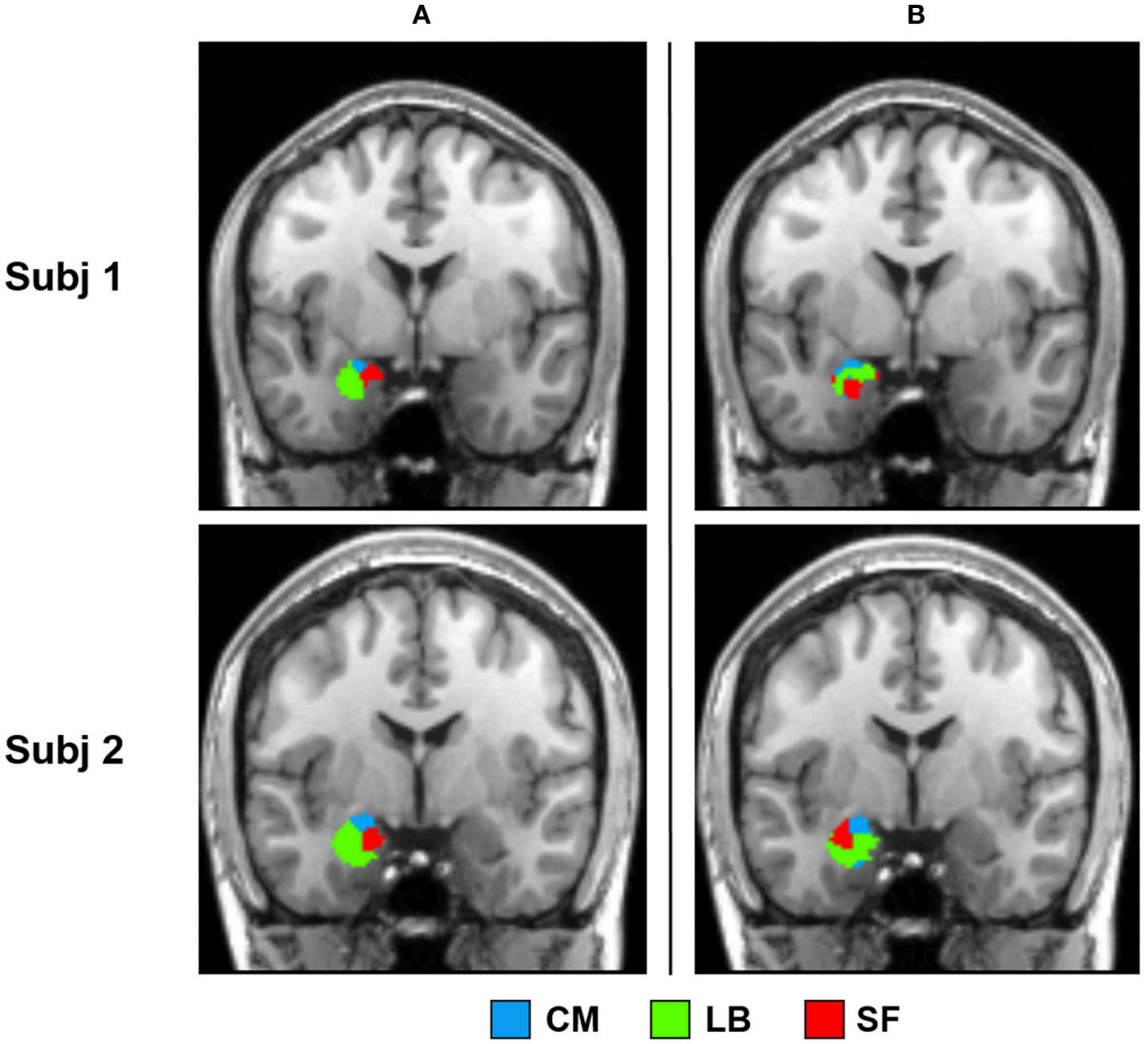
Comparison between the SSC partition and NCUT partition. **(a)** Parcellation results of two representative subjects obtained by the SSC method. **(b)** Parcellation results of the same two subjects obtained by the NCUT method. SSC, Semi-supervised clustering; NCUT, Normalized cut; CM, centromedial; LB, laterobasal; SF, superficial.

Parcellation results of two representative subjects using SSC and NCUT methods are shown in Figure 6. Visual inspection shows that the parcellation results generated by SSC method are more spatially continuous than the NCUT method. Besides, the average entropy of SSC parcellation results for left and right amygdala is 0.290 and 0.428, respectively. While the average entropy of NCUT parcellation results for left and right amygdala is 0.746 and 0.839, respectively, which is significantly higher than the SSC partition. The comparison results demonstrate that the SSC method has better performance than the NCUT method in terms of cross subject consistency because of adopting prior information to guide the functional parcellation. In addition, the overlap degree between the parcellation results generated by the SSC method and cytoarchitectonic map is higher than the overlap degree between the parcellation results generated by the NCUT method and cytoarchitectonic map (Table 2).

### Functional connectivity patterns of the amygdala parcellation results

#### FC patterns of the amygdala's subregions

**Figure 7:**
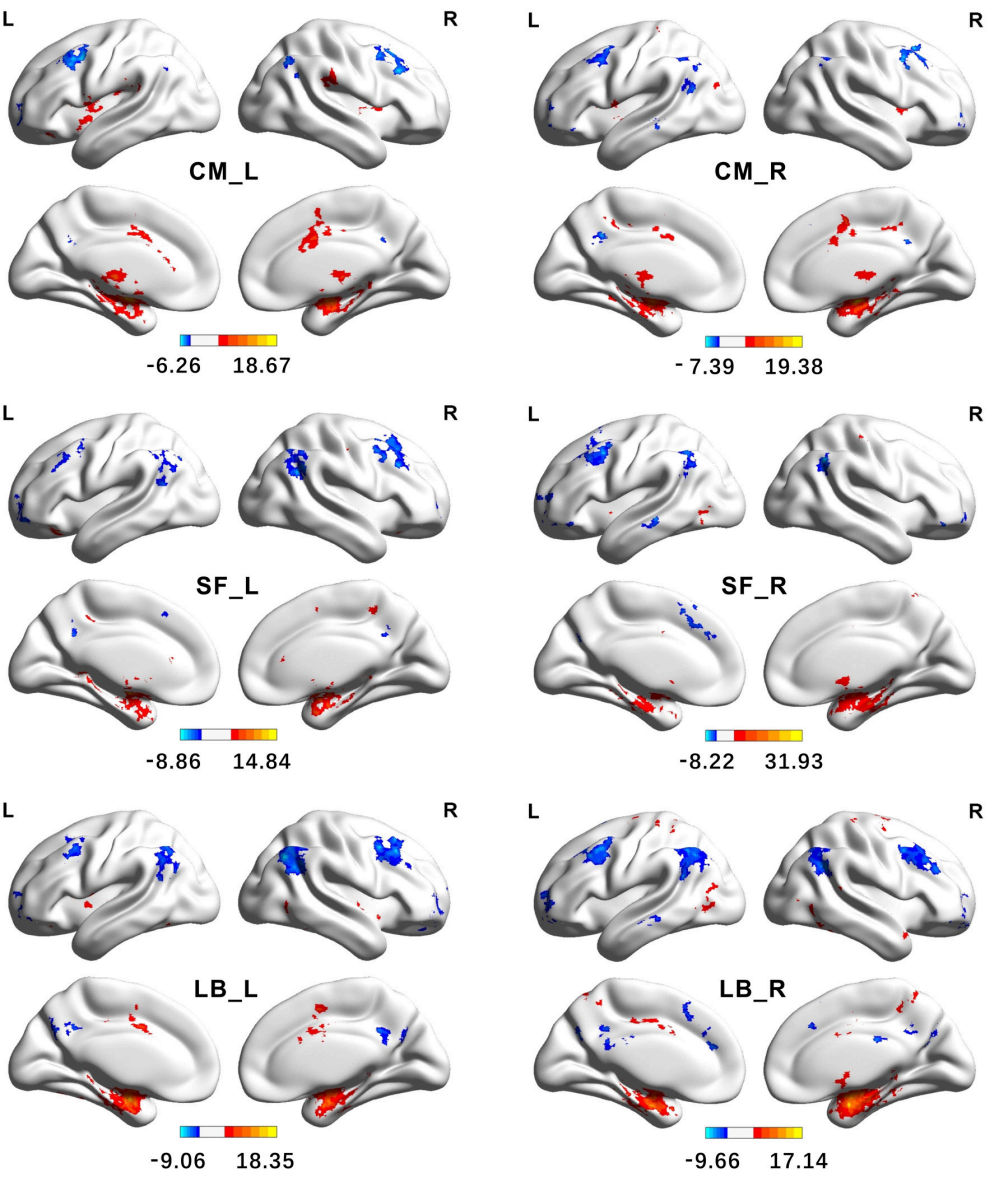
Functional Connectivity patterns of the amygdala's subregions identified using semi-supervised clustering method. The colorbar shows the *t*-values of one sample *t*-tests. Clusters were identified using one sample *t*-tests, significant at a threshold of *p* < 0.001 and an extent threshold of *p* < 0.05 with cluster-level family-wise error correction. CM, centromedial; LB, laterobasal; SF, superficial; L, left hemisphere; R, right hemisphere.

As shown in Figure 7 and Supplementary Tables 1–3, the group-level FC patterns are distinctive among the parcellated three amygdala subregions, which is described in detail as follows.

The brain regions, showing significantly positive FC with the left CM, are mainly located in the bilateral striatum, thalamus, insula, supramarginal gyrus, part of anterior and middle cingulate gyrus, and part of the cerebellum. The right CM has significantly positive FC with the bilateral putamen, pallidum, insula, middle cingulate gyrus, left precentral gyrus, left postcentral gyrus, right supplementary motor cortex, and part of the cerebellum. Conversely, the brain regions, showing significantly negative FC with the CM, are mainly located in the angular gyrus, precuneus, middle frontal gyrus, and part of the superior frontal gyrus.

The brain regions, showing significantly positive FC with the left LB, are primarily located in the hippocampus, parahippocampal gyrus, inferior and middle temporal gyrus, temporal pole, cingulate gyrus, left insula, precentral gyrus, and the fusiform gyrus. Besides these brain areas, the right LB also has extensive positive FC with bilateral brain regions including the precuneus, postcentral gyrus, and the precentral gyrus. On the contrary, the LB has significantly negative FC with the brain regions including the angular gyrus, precuneus, middle cingulate gyrus, medial frontal gyrus, middle and superior frontal gyrus.

The left SF has significantly positive FC with brain regions including the hippocampus, parahippocampus, pallidum, left anterior and middle cingulate gyrus, part of right precentral gyrus, and part of the cerebellum. The brain regions, showing extensive positive FC with the right SF, are the hippocampus, parahippocampus, left middle cingulate gyrus, part of right precentral gyrus, and right precuneus. Conversely, the SF has significantly negative FC with brain regions including the angular gyrus, middle frontal gyrus, inferior orbital frontal gyrus, part of the left precuneus, and part of the middle temporal gyrus.

#### FC difference between the ipsilateral amygdala subregions

The differences in FC patterns between the ipsilateral amygdala subregions are described as follows, which are shown in Tables 3–**5**.

**Table 3:** Function connectivity difference between centromedial (CM) and laterobasal (LB) amygdala subregion.

	**BA**	**x**	**y**	**z**	**z score**	**Cluster size**
**LEFT HEMISPHERE**						
***CM>LB***						
Amygdala(L)		−19.5	−4.5	−13.5	6.00	305
Thalamus(R)		18	−22.5	18	4.82	141
Pallidum(R)		18	1.5	−9	4.50	255
Midbrain		−1.5	−31.5	−15	4.47	71
Cerebellum Anterior Lobe(L)		−1.5	−69	−12	4.37	78
Putamen(R)		27	−7.5	12	4.37	87
Caudate(R)		16.5	−4.5	16.5	4.25	77
Caudate(L)		−12	1.5	12	4.24	343
***LB>CM***						
Amygdala(L)		−25.5	−1.5	−25.5	5.88	265
Middle occipital gyrus(L)	19	−34.5	−81	3	4.57	246
Fusiform gyrus(L)	37	−33	−61.5	−18	4.55	69
Fusiform gyrus(R)		25.5	−51	−18	4.48	92
Lingual gyrus(R)	18	19.5	−70.5	−6	4.27	91
Cerebellum posterior lobe		−22.5	−60	−15	3.93	75
**RIGHT HEMISPHERE**						
***CM>LB***						
Amygdala(R)		24	−9	−15	5.43	195
Caudate(R)		7.5	−10.5	10.5	4.63	232
Angular(L)	39	−39	−69	30	4.58	93
Thalamus(L)		−15	−13.5	13.5	4.42	246
Thalamus(R)		18	−19.5	4.5	4.41	125
Cerebellum posterior lobe		6	−73.5	−18	4.40	59
Thalamus(R)		21	−18	12	4.33	62
***LB>CM***						
Amygdala(R)		25.5	−1.5	−25.5	6.21	432
Middle occipital gyrus(L)	19	−40.5	−69	4.5	4.76	474
Fusiform gyrus(R)	18	27	−75	−9	4.37	125
Superior parietal gyrus(L)		−36	−57	63	4.23	226
Inferior parietal gyrus(L)	2	−54	−27	46.5	4.15	107
Middle occipital gyrus(R)		39	−76.5	13.5	4.13	97
Postcentral(R)	2	40.5	−40.5	54	3.98	137
Inferior occipital lobe(L)		−37.5	−75	−10.5	3.93	79
Fusiform gyrus(L)		−24	−70.5	−9	3.90	62

*All clusters are significant at a threshold of p < 0.001 and an extent threshold of p < 0.05 with cluster-level family-wise error correction. Secondary local maxima within the significant clusters are not listed*.

The CM has significantly stronger positive FC with the striatum, thalamus, and part of the cerebellum anterior lobe than the ipsilateral LB or SF subregions as shown in Tables 3, 4.

**Table 4:** Function connectivity difference between centromedial (CM) and superficial (SF) amygdala subregion.

	**BA**	**x**	**y**	**z**	**Z score**	**Cluster size**
**LEFT HEMISPHERE**						
***CM>SF***						
Amygdala(L)		−24	−4.5	−13.5	6.11	1,905
Pallidum(L)		−22.5	−3	1.5	5.71	
Pallidum(R)		24	−4.5	4.5	5.71	1,452
Putamen(R)		31.5	−9	−10.5	5.35	
Thalamus(R)		15	−18	0	4.42	61
Cerebellum anterior lobe		4.5	−58.5	−27	4.19	76
***SF>CM***						
Amygdala(L)		−18	−4.5	−19.5	5.62	69
Precuneus(R)		25.5	−61.5	24	4.35	108
Cerebellum Anterior Lobe		−13.5	−43.5	−12	4.25	79
Calcarine (L)		−18	−63	18	4.15	76
Lingual(R)	18	18	−75	−3	4.14	105
**RIGHT HEMISPHERE**						
***CM>SF***						
Amygdala(R)		25.5	−6	−13.5	6.22	356
Putamen(R)		30	−7.5	0	3.97	
Cerebellum anterior lobe		6	−40.5	−25.5	4.70	74
Temporal lobe(L)		–−33	−9	−9	4.67	270
Putamen(L)		−33	−16.5	−4.5	4.36	
Thalamus(R)		15	−19.5	3	4.50	95
Middle frontal gyrus(L)	6	−24	3	58.5	4.01	74
Thalamus(L)		−9	−9	3	3.83	65
***SF>CM***						
Parahippocampus(R)		18	−6	−18	6.28	195
Superior parietal gyrus(L)		−25.5	−51	48	5.35	68
Middle occipital gyrus(R)		33	−82.5	13.5	4.77	176
Middle occipital gyrus(L)	19	−33	−88.5	3	4.46	647
Middle occipital gyrus(L)		−30	−79.5	4.5	4.32	
Lingual(L)		−22.5	−63	−10.5	4.33	72
Middle occipital gyrus(R)	19	36	−87	3	4.20	92
Inferior occipital lobe(L)	18	−34.5	−82.5	−12	4.15	124
Inferior parietal lobule(R)	7	27	−55.5	51	4.12	59
Fusiform gyrus(L)	18	−21	−76.5	−16	3.91	59

*All clusters are significant at a threshold of p < 0.001 and an extent threshold of p < 0.05 with cluster–level family–wise error correction. Secondary local maxima within the significant clusters are not listed*.

As shown in Table 3, the left LB has significantly stronger FC with the bilateral fusiform gyrus, and part of the left middle occipital lobe than the left CM, while the right LB has higher FC with the bilateral fusiform gyrus, part of the bilateral middle occipital lobe, left inferior occipital lobe, left superior parietal lobe, and part of the right postcentral gyrus than the right CM. Compared with the SF, the LB has higher FC with the hippocampus, parahippocampus, middle temporal lobe, and part of the precentral gyrus (Table 5).

**Table 5:** Function connectivity difference between laterobasal (LB) and superficial (SF) amygdala subregion.

	**BA**	**x**	**y**	**z**	**Z score**	**Cluster size**
**LEFT HEMISPHERE**						
**LB>SF**						
Hippocampus(L)		−27	−7.5	−22.5	5.94	351
Hippocampus(R)		27	−9	−25.5	4.77	131
Middle temporal gyrus(L)	19	−49.5	−60	−3	4.58	62
Precentral(L)		−52.5	3	19.5	4.35	102
***SF>LB***						
Putamen(L)		−9	7.5	−9	5.14	119
Caudate(R)		7.5	12	−3	4.43	79
Precuneus(R)	7	4.5	−70.5	45	4.17	66
**RIGHT HEMISPHERE**						
***LB>SF***						
Parahippocampus(R)		31.5	−1.5	−25.5	5.59	393
SupraMarginal(L)		−57	−36	25.5	5.05	55
Hippocampus(L)		−27	−7.5	−24	4.86	74
Precentral(L)	6	−19.5	−15	67.5	4.82	226
Middle temporal gyrus(L)	19	−51	−66	13.5	3.76	54
***SF>LB***						
Amygdala(R)		18	−6	−18	5.86	88
Middle cingulum gyrus(L)		−9	−42	34.5	4.46	54
Precuneus(L)		−7.5	−81	40.5	4.04	73

*All clusters are significant at a threshold of p < 0.001 and an extent threshold of p < 0.05 with cluster-level family-wise error correction. Secondary local maxima within the significant clusters are not listed*.

The SF has significantly higher FC with part of the occipital gyrus and the lingual gyrus than the CM (Table 4). Compared with the LB, the SF has higher FC with the striatum, the middle cingulate gyrus, and part of the precuneus (Table 5).

#### Asymmetry in FC of the bilateral amygdala subregions

**Figure 8:**
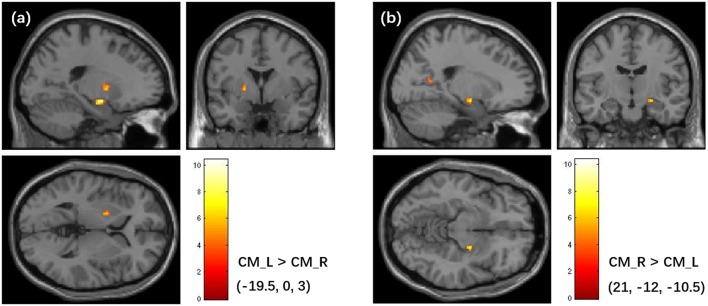
Asymmetry in FC patterns of the left CM amygdala and right CM amygdala. **(a)** Brain regions that shows higher FC with the left CM than the right CM. **(b)** Brain regions that shows higher FC with the right CM than the left CM. The colorbar shows the *t*-values of paired *t*-tests. The clusters are identified using the paired *t*-test, significant at a threshold of *p* < 0.001 and an extent threshold of *p* < 0.05 with cluster-level family-wise error correction. FC, Functional Connectivity; CM, centromedial.

The asymmetry in FC of the bilateral amygdala's subregions was found in this study. Particularly, the left and right CM amygdala have significant difference in FC patterns (Figure 8), while no significant difference was found in bilateral LB amygdala and bilateral SF amygdala (*P* < 0.05, cluster level FWE correction). The left CM has higher FC with the left pallidum than the right CM, while the right CM has higher FC with the right hippocampus and the posterior cingulate cortex than the left CM.

## Discussion

In this study, we proposed a semi-supervised clustering based brain parcellation scheme for segmenting human amygdala into functionally homogeneous subregions at an individual level. The proposed method successfully parcellated human amygdala into 3 subregions in every subject, namely the CM, LB, and SF group, based on connectivity structure of a 7T high-resolution rs-fMRI dataset. Volume measurements and subregion probability map analyses have demonstrated that our method is capable to effectively capture the inter-subject variability in distribution of amygdala functional subregions. Besides, functional homogeneity of our parcellation results is better than that of the cytoarchitectonic atlas, as validated by the paired *t*-tests of silhouette width index. More importantly, the obtained amygdala subregions are characterized by specific functions, which is evidenced by their distinctive and asymmetric FC patterns.

### Methodological consideration of the SSC method

In the noninvasive studies for exploring human amygdala subregional function or connectivity using fMRI technique, a prerequisite step is specifying functionally distinct subregions of amygdala. Previous studies have applied different strategies to define the amygdala subregions, including the use of cytoarchitectonic or anatomical atlas (Amunts et al., 2005; Saygin et al., 2017), clustering neuroimaging data with unsupervised algorithm (Bach et al., 2011; Solano-Castiella et al., 2011; Zarei et al., 2013; Mishra et al., 2014; Tyszka and Pauli, 2016). The proposed method in this study is a little different from previous strategies.

Firstly, compared with the cytoarchitectonic atlas, the proposed method is equipped to capture the inter-subject variability in distribution of functional subregions, which is consistent with the brain parcellation approaches in previous important studies (Blumensath et al., 2013; Wang et al., 2015, 2017). Obtaining functional mapping of brain area at the level of the individual is a critical step toward understanding the association between anatomy and function in the human brain. Though the cytoarchitectonic atlas has been widely used in fMRI studies, one of their significant limitations is that they identify amygdala subregions at a group level. Our method offers an alternative approach to identify amygdala functional subregions at an individual subject level. As a result, substantial inter-individual variability is captured in the parcellation results. The obtained subregions have visually different distributions in every subject (Figure 2). The volume measurement results and probability map of each subregion further confirm that there is a considerable variability in size and distribution for each amygdala subregion between different subjects (Figure 3, Table 1).

Secondly, the proposed SSC based brain parcellation method is different from the unsupervised clustering based brain parcellation methods because it can incorporate prior information that is derived from the existing neuroanatomy knowledge (i.e., cytoarchitectonic atlas) to guide the brain parcellation. Actually, in many studies unsupervised clustering methods are used to parcellate brain area based on tractography (Wang et al., 2012), resting-state FC (Wang et al., 2017), multi-contrast structure image (Solano-Castiella et al., 2011), and brain-wide coactivation map (Bzdok et al., 2013). In these studies, cytoarchitectonic mapping information is commonly used to determine the number of clusters or used to validate the parcellation results by regarding the cytoarchitectonic parcellation as the golden standard. In a different way, we directly use the prior information derived from the cytoarchitectonic map to guide the functional parcellation, thus facilitating an anatomically and functionally consistent parcellation of the human amygdala. Unsupervised clustering methods have been widely used to parcellate brain areas for individual subjects (Mishra et al., 2014; Wang et al., 2017). However, these methods do not guarantee the individual parcellation results directly comparable across subjects, parcellation results of different subjects have to be matched somehow to establish correspondence across subjects. They need postprocessing steps to define the amygdala subregions in common anatomical space (Mishra et al., 2014). But the proposed SSC brain parcellation method could generate subject specific brain parcellation results without losing correspondence across subjects, thus having a higher cross subject consistency of partitioning. Besides, the SSC method can keep good concordance with the anatomical space because of the prior information derived from the widely accepted cytoarchitectonic atlas (Table 2). As shown in Figures 3D,E, the functional MPM of SSC parcellation also shares similarity with MPM of the Julich atlas albeit different. Furthermore, the comparison results have demonstrated that functional homogeneity of the SSC parcellation is better than the Julich atlas (Figure 5). Besides, Mishra et al. (2014) clustered voxels in amygdala mainly based on the similarity between whole brain FC maps associated with each voxel, while our method clustered voxels in amygdala mainly based on the similarity of timecourses extracted from these voxels, which may have a higher computing efficiency and help to achieve better temporal homogeneity within a cluster (Craddock et al., 2012). It's noteworthy that the Julich atlas is not directly used as prior information, only three small regions with most homogeneous functional signals are extracted from the Julich atlas and adopted as prior information. Thus, it's reasonable to compare the SSC parcellation with Julich atlas in terms of functional homogeneity. Besides, the weighting of anatomical priors in the objective function is carefully determined by reference to Cheng and Fan (2014). A discussion about how spatial constraints affect the functional parcellation results can be found in Craddock et al. (2012) and Cheng et al. (2014).

### Functional connectivity of the obtained subregions

Functional subregions obtained by the proposed SSC method (namely the CM, LB, and SF group) may involve in different functions and networks, which is evidenced by the whole brain FC analysis.

While most of our findings about the amygdala subregional connectivity patterns are in accordance with prior work (Roy et al., 2009; Qin et al., 2014; Engman et al., 2016), from a methodological perspective, the applied 7T high resolution fMRI data can guarantee the spatial fidelity of fMRI signals, therefore allowing a more accurate definition of amygdala subregions as well as their FC patterns. Recent technical advances in ultrahigh field MRI, such as 7T, have made it possible to acquire high quality MRI data with linearly increased signal to noise ratio (SNR). The SNR gains can bring higher spatial resolution, which will improve the spatial fidelity of the functional signals and increase the functional contrast (Ugurbil et al., 2013). Considering the total volume of human amygdala is just around 2 cm^3^ (Brabec et al., 2010), a high resolution 7T rs-fMRI dataset will greatly improve the spatial specificity of the parcellation results.

We found the CM had significantly positive FC with the striatum, thalamus, insula, middle cingulate gyrus, and part of the cerebellum, which were in line with the previous studies (Roy et al., 2009; Bzdok et al., 2013). These findings supported the CM's role as the amygdala's major output center mediating both motor responses and autonomic responses (Pitkänen et al., 1997; Pessoa, 2011).

Our results showed the LB had extensive positive connectivity with the hippocampus, parahippocampus, the inferior and middle temporal gyrus, temporal pole, precentral gyrus, and the fusiform gyrus, which corresponded well with the findings based on the 3T rs-fMRI data in the previous studies (Roy et al., 2009; Mishra et al., 2014). The findings supported the argument that the laterobasal nucleus might be implicated in significance detection and associative learning processes (Phillips et al., 2003; Phelps and LeDoux, 2005; Roy et al., 2009; Bzdok et al., 2013). Further comparison between the ipsilateral LB and CM found the LB had stronger FC with the fusiform gyrus and the inferior occipital gyrus, which supported its role in processing high-level visual input. The finding was corroborated by previous studies on monkeys (Iwai and Yukie, 1987) and the meta-analytic coactivation study on human (Bzdok et al., 2013). Besides, the LB also had significantly negative FC with the angular gyrus, medial and superior frontal gyrus, orbital frontal gyrus, and the precuneus, which might be consistent with the proposition of a similar network involved in emotion regulation in the previous studies (Phillips et al., 2003; Blair et al., 2007; Roy et al., 2009).

The SF amygdala, which was thought to play an important role in olfaction input processing in animals before (Kemppainen et al., 2002; Moreno and González, 2007), has recently been revealed to also involve in processing social information such as olfactory and emotional stimuli in human (Hurlemann et al., 2008; Goossens et al., 2009). In this study, the SF had stronger FC with the striatum and the middle cingulate gyrus than the LB, and had stronger FC with part of the occipital gyrus and the lingual gyrus than the CM, which accorded well with the SF's important role in the social interaction.

The asymmetry in FC was found between the left and right CM. Specifically, the left CM had higher FC with the left pallidum, and the right CM had higher FC with the right hippocampus, and the posterior cingulate cortex. Although no significant difference was found for the LB and SF subregions at the significance level of *P* < 0.05 (cluster level FWE correction), we found LB and SF showed higher connectivity with distinct ipsilateral brain areas if the significance level was set to *P* < 0.001 (uncorrected). Prior work has revealed that there was FC difference between left and right hemispheres for each amygdala subregion defined using the SPM Anatomy toolbox (Kerestes et al., 2017), which was generally consistent with the findings in this study. The asymmetry in FC of the CM partially supported the difference in functions between the left and right amygdala, which has been revealed by the previous studies (Baas et al., 2004; Hardee et al., 2008; Polli et al., 2009).

One limitation of the present study is typical in researches that utilize fMRI technique to investigate amygdala function. The amygdala is located in a brain area that is easily affected by inhomogeneity of B_0_ field. The GRE-EPI will show considerable image distortion and signal dropouts in the amygdala due to intra-voxel dephasing. However, the negative effects of intra-voxel dephasing can be efficiently compensated by reducing voxel sizes, i.e., increased in-plane resolution and/or thinner slices. This strategy has been used by a number of fMRI studies at a field of 3 Tesla or 7 Tesla (Morawetz et al., 2008, 2016; Hahn et al., 2011; Sladky et al., 2015, 2017). In this study, careful manually shimming of B_0_ field was performed and high spatial resolution acquisition protocol (resolution: 1.5 mm isotropic) was used, which has efficiently reduced the intra-voxel dephasing and decreased the image distortion in amygdala area. Another limitation of this study is that the sample size is a little small. Nevertheless, as discussed above, the amygdala subregional FC patterns obtained by our method were primarily consistent with previous studies.

## Conclusion

In conclusion, the presented semi-supervised spectral clustering based brain parcellation method can successfully parcellate the human amygdala into three functionally homogeneous and spatially coherent subregions at an individual subject level. Furthermore, validation experiments have revealed that these subregions are characterized by distinct FC patterns. Our study has demonstrated that the semi-supervised brain parcellation method can serve as a powerful tool in fMRI studies for investigating subregional functions of human amygdala.

## Author contributions

XZ, ZZ, RX and YF conceived and designed the study; XZ, HC and YF developed the parcellation method; XZ, ZZ and RX designed the experiments and analyzed the fMRI data; KZ, FC, BW cooperated to collect and analyze data; YZ and LC managed the imaging platform and helped experimental design; XZ and HC drafted the manuscript; YF, RX and ZZ modified and proofread the manuscript; XZ and HC contributed equally to this work; YF provided the project idea.

### Conflict of interest statement

The authors declare that the research was conducted in the absence of any commercial or financial relationships that could be construed as a potential conflict of interest.

## Abbreviations

fMRI: functional magnetic resonance imaging
rs-fMRI: resting state functional magnetic resonance imaging
sMRI: structural MRI
DTI: diffusion tensor imaging
GRE-EPI: gradient recalled echo planar imaging
T1w: T1-weighted
MPRAGE: magnetization-prepared rapid gradient-echo
TR/TE/TI: time repetition/echo time/inversion time
FA: flip angle
FOV: field of view
iPAT: intergrated parallel acquisition technique
MNI: Montreal Neurologic Institute
SSC: semi-supervised spectral clustering
MPM: maximum probability map
SI: silhouette width
ROI: region of interest
CM: centromedial
LB: laterobasal
SF: superficial.
