# Supplementary material for: Individualized Functional Parcellation of the Human Amygdala Using a Semi-supervised Clustering Method: A 7T Resting State fMRI Study
Source: Front Neurosci. 2018 Apr 26;12:270. doi: 10.3389/fnins.2018.00270 (PMC5932177; doi:10.3389/fnins.2018.00270)
Supplement: Supplementary file 1 [file Table_1.DOCX]

**TABLE I**. Function connectivity pattern of centromedial(CM) amygdala.

|  | BA | x | y | z | z  score | cluster size |
| --- | --- | --- | --- | --- | --- | --- |
| ***Left Hemisphere*** |  |  |  |  |  |  |
| ***Positive Connectivity*** |  |  |  |  |  |  |
| Amygdala(L) |  | -25.5 | -6 | -13.5 | 7.43 | 14866 |
| Amygdala(R) |  | 25.5 | -3 | -15 | 6.96 |  |
| Frontal_Mid_Orb_L | 11 | -24 | 31.5 | -16.5 | 5.73 | 223 |
| Cerebellum Posterior Lobe(R) |  | 1.5 | -67.5 | -18 | 5.48 | 801 |
| Middle Cingulum Gyrus(L) | 24 | 0 | 3 | 37.5 | 5.23 | 1698 |
| SupraMarginal(L) |  | -60 | -27 | 24 | 4.70 | 247 |
| SupraMarginal(R) |  | 60 | -27 | 30 | 4.51 | 351 |
| Cerebellum Anterior Lobe(R) |  | 22.5 | -46.5 | -25.5 | 4.47 | 221 |
| Superior Temporal Gyrus(R) | 38 | 48 | 15 | -9 | 4.29 | 191 |
| Rolandic_Oper_R | 22 | 61.5 | 4.5 | 4.5 | 4.18 | 107 |
| Cerebellum_4_5_R |  | 9 | -48 | -12 | 4.16 | 70 |
| Insula(L) |  | -36 | -18 | 16.5 | 4.04 | 82 |
| Anterior Cingulum Gyrus(L) |  | -1.5 | 24 | 16.5 | 4.02 | 76 |
|  |  |  |  |  |  |  |
| **Negative Connectivity** |  |  |  |  |  |  |
| Middle Frontal Gyrus(L) | 6 | -30 | 12 | 57 | 4.56 | 974 |
| Precuneus(R) | 31 | 13.5 | -49.5 | 31.5 | 4.48 | 86 |
| Angular(L) |  | -40.5 | -52.5 | 24 | 4.48 | 104 |
| Middle Frontal Gyrus(R) | 8 | 36 | 18 | 48 | 4.45 | 1021 |
| Precuneus(L) |  | -15 | -58.5 | 31.5 | 4.43 | 118 |
| Frontal_Mid_Orb_L | 10 | -30 | 51 | -3 | 4.34 | 417 |
| Angular(L) |  | -43.5 | -60 | 37.5 | 4.08 | 78 |
| Angular(R) |  | 42 | -60 | 42 | 3.96 | 233 |
|  |  |  |  |  |  |  |
| ***Right Hemisphere*** |  |  |  |  |  |  |
| **Positive Connectivity** |  |  |  |  |  |  |
| Amygdala(R) |  | 24 | -9 | -15 | 7.52 | 9758 |
| Amygdala(L) |  | 6 | 0 | 46.5 | 5.33 | 895 |
| Thalamus(L) | 24 | -16.5 | -15 | 13.5 | 4.96 | 134 |
| Supp_Motor_Area_R |  | 12 | -6 | 76.5 | 4.84 | 183 |
| Insula(R) | 38 | 46.5 | 16.5 | -15 | 4.79 | 548 |
| Midbrain |  | -1.5 | -19.5 | -13.5 | 4.62 | 63 |
| Middle Cingulum Gyrus(R) |  | 7.5 | -40.5 | 45 | 4.61 | 215 |
| Middle Cingulum Gyrus(L) |  | -12 | -39 | 45 | 4.52 | 232 |
| Cerebellum_3_R |  | 9 | -42 | -22.5 | 4.39 | 142 |
| Precentral(L) |  | -19.5 | -21 | 78 | 4.36 | 131 |
| Precuneus(R) |  | 18 | -52.5 | 12 | 4.15 | 90 |
| Postcentral Gyrus(L) | 3 | -21 | -33 | 54 | 4.13 | 113 |
| ParaHippocampal(L) |  | -30 | -42 | -10.5 | 4.06 | 76 |
| Midbrain |  | 4.5 | -33 | -12 | 3.93 | 77 |
| Middle Temporal Gyrus(L) |  | -34.5 | -73.5 | 22.5 | 3.86 | 74 |
|  |  |  |  |  |  |  |
| **Negative Connectivity** |  |  |  |  |  |  |
| Middle Frontal Gyrus(R) | 8 | 22.5 | 16.5 | 52.5 | 5.01 | 110 |
| Middle Frontal Gyrus(L) |  | -40.5 | 7.5 | 52.5 | 4.88 | 611 |
| Middle Cingulum Gyrus(L) | 31 | -12 | -48 | 36 | 4.81 | 208 |
| Precentral(R) |  | 42 | 7.5 | 43.5 | 4.74 | 592 |
| Superior Temporal Gyrus(L) |  | -54 | -57 | 19.5 | 4.59 | 343 |
| Middle Frontal Gyrus(R) |  | 37.5 | 55.5 | 0 | 4.46 | 252 |
| Precuneus(R) | 7 | 3 | -54 | 39 | 4.38 | 105 |
| Medial Frontal Gyrus(L) |  | -13.5 | 16.5 | 51 | 4.37 | 143 |
| Inferior Parietal Lobule(R) | 40 | 45 | -54 | 40.5 | 4.25 | 418 |
| Middle Temporal Gyrus(L) |  | -57 | -37.5 | -4.5 | 4.22 | 229 |
| Superior Frontal Gyrus(R) |  | 16.5 | 22.5 | 52.5 | 4.20 | 72 |
| Middle Frontal Gyrus(L) | 10 | -37.5 | 55.5 | 7.5 | 4.15 | 302 |
| Inferior Frontal Gyrus(L) |  | -46.5 | 33 | -10.5 | 4.07 | 74 |
| Angular(L) | 39 | -37.5 | -64.5 | 34.5 | 3.87 | 99 |
| Inferior Frontal Gyrus(L) | 45 | -48 | 18 | 3 | 3.86 | 77 |
| Inferior Parietal Lobule(L) | 40 | -49.5 | -52.5 | 45 | 3.79 | 195 |

All clusters are significant at a threshold of $p<0.001$ and an extent threshold of $p<0.05$ with cluster-level family-wise error correction. Secondary local maxima within the significant clusters are not listed.
