# Supplementary material for: Individualized Functional Parcellation of the Human Amygdala Using a Semi-supervised Clustering Method: A 7T Resting State fMRI Study
Source: Front Neurosci. 2018 Apr 26;12:270. doi: 10.3389/fnins.2018.00270 (PMC5932177; doi:10.3389/fnins.2018.00270)
Supplement: Supplementary file 2 [file Table_2.DOCX]

**TABLE II**. Function connectivity pattern of laterobasal(LB) amygdala.

|  | BA | x | y | z | z score | cluster size |
| --- | --- | --- | --- | --- | --- | --- |
| ***Left Hemisphere*** |  |  |  |  |  |  |
| ***Positive Connectivity*** |  |  |  |  |  |  |
| Hippocampus(L) |  | -24 | -6 | -25.5 | 7.39 | 3459 |
| Hippocampus(R) |  | 27 | -6 | -22.5 | 6.65 | 2775 |
| Cingulate Gyrus(R) |  | 9 | 1.5 | 49.5 | 5.21 | 957 |
| Fusiform Gyrus(L) |  | -34.5 | -45 | -24 | 5.14 | 242 |
| Fusiform Gyrus(R) |  | 25.5 | -49.5 | -18 | 4.59 | 238 |
| Parahippocampa Gyrus(R) |  | 13.5 | -34.5 | -3 | 4.55 | 66 |
| Temporal_Pole_Sup_L |  | -49.5 | 13.5 | -16.5 | 4.25 | 64 |
| Fusiform Gyrus(R) |  | 46.5 | -45 | -18 | 4.22 | 116 |
| Middle Temporal Gyrus(R) | 37 | 48 | -63 | -1.5 | 4.21 | 73 |
| Middle Cingulum Gyrus(L) |  | -9 | -24 | 45 | 4.20 | 98 |
| Insula(L) |  | -36 | -6 | 9 | 4.09 | 72 |
| ParaHippocampal(L) |  | -16.5 | -36 | -3 | 4.03 | 72 |
| Precentral Gyrus(L) |  | -52.5 | 0 | 7.5 | 3.97 | 123 |
| Middle Frontal Gyrus(L) |  | -22.5 | 31.5 | -15 | 3.81 | 64 |
|  |  |  |  |  |  |  |
| ***Negative Connectivity*** |  |  |  |  |  |  |
| Angular(R) |  | 36 | -48 | 25.5 | 5.57 | 2746 |
| Middle Frontal Gyrus(L) |  | -39 | 13.5 | 42 | 5.29 | 768 |
| Middle Frontal Gyrus(R) |  | 40.5 | 9 | 45 | 5.20 | 1721 |
| Angular(L) | 40 | -48 | -61.5 | 45 | 5.03 | 1389 |
| Precuneus(L) |  | -12 | -55.5 | 25.5 | 4.73 | 721 |
| Frontal_Sup_Orb_L |  | -30 | 54 | -1.5 | 4.58 | 596 |
| Superior Frontal Gyrus(R) |  | 21 | 45 | -3 | 4.57 | 661 |
| Cuneus(L) |  | 1.5 | -75 | 33 | 4.48 | 984 |
| Medial Frontal Gyrus(R) |  | 15 | 55.5 | 16.5 | 4.08 | 88 |
| Middle Temporal Gyrus(L) | 21 | -66 | -24 | -9 | 4.01 | 62 |
| Superior Frontal Gyrus(R) | 11 | 19.5 | 52.5 | -16.5 | 3.81 | 77 |
|  |  |  |  |  |  |  |
| ***Right Hemisphere*** |  |  |  |  |  |  |
| ***Positive Connectivity*** |  |  |  |  |  |  |
| Amygdala(R) |  | 24 | -1.5 | -21 | 7.22 | 3858 |
| Amygdala(L) |  | -21 | -6 | -18 | 6.55 | 2711 |
| Middle Temporal Gyrus(L) |  | 51 | -64.5 | -1.5 | 4.94 | 277 |
| Precuneus(L) | 7 | -10.5 | -64.5 | 66 | 4.87 | 403 |
| Cingulate Gyrus(R) | 24 | 3 | -13.5 | 42 | 4.79 | 627 |
| Fusiform Gyrus(R) | 37 | 42 | -49.5 | -21 | 4.78 | 152 |
| Insula(L) |  | -34.5 | -4.5 | 9 | 4.73 | 81 |
| Temporal_Pole_Mid_R | 38 | 52.5 | 9 | -24 | 4.56 | 66 |
| Postcentral(L) |  | -48 | -33 | 61.5 | 4.55 | 99 |
| Middle Frontal Gyrus(R) | 6 | 24 | -9 | 66 | 4.54 | 200 |
| Precentral(L) | 6 | -19.5 | -12 | 72 | 4.53 | 166 |
| Superior Parietal Gyrus(L) |  | -19.5 | -52.5 | 75 | 4.44 | 95 |
| Postcentral(L) |  | -27 | -37.5 | 73.5 | 4.41 | 287 |
| Superior Temporal Gyrus(L) |  | -37.5 | -12 | -9 | 4.39 | 165 |
| Precuneus(R) | 7 | 6 | -54 | 64.5 | 4.37 | 231 |
| Middle Temporal Gyrus(L) |  | -46.5 | -72 | 12 | 4.34 | 137 |
| Cerebelum_6_L |  | -39 | -49.5 | -25.5 | 4.27 | 127 |
| Lingual_R |  | 15 | -36 | -1.5 | 4.26 | 74 |
| Precentral(R) |  | 27 | -28.5 | 73.5 | 4.25 | 144 |
| Middle Temporal Gyrus(L) |  | -42 | -61.5 | 6 | 4.25 | 289 |
| Superior Temporal Gyrus(R) |  | 63 | -42 | 10.5 | 4.20 | 85 |
| Caudate Head |  | 6 | 3 | 0 | 4.18 | 128 |
| Precuneus(L) | 7 | -13.5 | -45 | 52.5 | 4.04 | 110 |
| Superior Temporal Gyrus(L) | 44 | -49.5 | 0 | 4.5 | 3.91 | 57 |
|  |  |  |  |  |  |  |
| ***Negative Connectivity*** |  |  |  |  |  |  |
| Middle Frontal Gyrus(R) |  | 46.5 | 28.5 | 34.5 | 5.74 | 1399 |
| Cuneus(L) | 7 | 0 | -76.5 | 33 | 5.60 | 1114 |
| Angular(L) | 7 | -40.5 | -61.5 | 52.5 | 5.59 | 2642 |
| Angular(R) |  | 52.5 | -57 | 33 | 5.38 | 1785 |
| Middle Frontal Gyrus(L) |  | -40.5 | 16.5 | 43.5 | 5.27 | 1635 |
| Middle Temporal Gyrus(L) |  | -52.5 | -25.5 | -15 | 5.20 | 176 |
| Middle Frontal Gyrus(L) |  | -30 | 54 | 0 | 5.00 | 1246 |
| undefined |  | 3 | -22.5 | 25.5 | 4.77 | 166 |
| Medial Frontal Gyrus | 9 | -9 | 39 | 21 | 4.76 | 80 |
| Middle Cingulum Gyrus(R) | 32 | 4.5 | 28.5 | 36 | 4.49 | 623 |
| Precuneus(R) |  | 16.5 | -63 | 22.5 | 4.46 | 147 |
| Frontal_Inf_Orb_R |  | 43.5 | 46.5 | -15 | 4.31 | 96 |
| Caudate Body |  | 13.5 | -6 | 21 | 4.13 | 74 |
| Superior Frontal Gyrus(L) |  | -13.5 | 7.5 | 64.5 | 4.11 | 93 |
| Middle Cingulum Gyrus(R) | 31 | 16.5 | -46.5 | 34.5 | 4.09 | 202 |
| Middle Frontal Gyrus(R) | 11 | 27 | 52.5 | -9 | 4.00 | 467 |

All clusters are significant at a threshold of $p<0.001$ and an extent threshold of $p<0.05$ with cluster-level family-wise error correction. Secondary local maxima within the significant clusters are not listed.
