# Supplementary material for: Individualized Functional Parcellation of the Human Amygdala Using a Semi-supervised Clustering Method: A 7T Resting State fMRI Study
Source: Front Neurosci. 2018 Apr 26;12:270. doi: 10.3389/fnins.2018.00270 (PMC5932177; doi:10.3389/fnins.2018.00270)
Supplement: Supplementary file 3 [file Table_3.DOCX]

**TABLE III**. Function connectivity pattern of superficial (SF) amygdala.

|  | BA |  | x | y | z score | cluster size |
| --- | --- | --- | --- | --- | --- | --- |
| ***Left Hemisphere*** |  |  |  |  |  |  |
| **Positive Connectivity** |  |  |  |  |  |  |
| Amygdala |  | -18 | -6 | -16.5 | 6.87 | 4462 |
| Cerebelum_4_5_L |  | -9 | -40.5 | -4.5 | 5.23 | 494 |
| Frontal_Inf_Orb_R |  | 31.5 | 33 | -18 | 4.77 | 98 |
| ParaHippocampas(R) |  | 21 | -42 | -7.5 | 4.68 | 128 |
| Temporal_Pole_Sup_R | 38 | 46.5 | 19.5 | -21 | 4.66 | 82 |
| Precentral(R) | 6 | 46.5 | -12 | 60 | 4.63 | 169 |
| Precentral(R) | 3 | 28.5 | -27 | 75 | 4.54 | 172 |
| Pallidum(R) |  | 27 | -18 | -4.5 | 4.31 | 75 |
| Postcentral(L) | 2 | -12 | -37.5 | 79.5 | 4.19 | 67 |
| Middle Cingulum Gyrus(L) | 24 | -1.5 | -4.5 | 37.5 | 4.18 | 149 |
| Middle Cingulum Gyrus(L) |  | -12 | -37.5 | 45 | 4.09 | 62 |
| Precuneus(R) | 5 | 10.5 | -42 | 54 | 4.09 | 87 |
| Anterior Cingulum Gyrus(L) | 24 | 1.5 | 34.5 | 12 | 4.04 | 80 |
| **Negative Connectivity** |  |  |  |  |  |  |
| Middle Frontal Gyrus_R |  | 45 | 25.5 | 34.5 | 5.51 | 1573 |
| Angular(R) |  | 51 | -55.5 | 28.5 | 5.16 | 2207 |
| Superior Frontal Gyrus(L) | 10 | -30 | 63 | 6 | 4.81 | 726 |
| Middle Frontal Gyrus(L) | 6 | -30 | 9 | 57 | 4.73 | 770 |
| Inferior Parietal Gyrus(L) | 40 | -52.5 | -46.5 | 49.5 | 4.62 | 922 |
| Middle Frontal Gyrus | 11 | 25.5 | 48 | -9 | 4.39 | 122 |
| Precuneus(L) | 31 | -3 | -49.5 | 37.5 | 4.37 | 418 |
| Supp_Motor_Area_L |  | -3 | 21 | 49.5 | 3.96 | 78 |
| Middle Frontal Gyrus(R) | 10 | 33 | 60 | -1.5 | 3.80 | 124 |
|  |  |  |  |  |  |  |
| ***Right Hemisphere*** |  |  |  |  |  |  |
| **Positive Connectivity** |  |  |  |  |  |  |
| Amygdala(R) |  | 20 | -6 | -18 | >8 | 3270 |
| Amygdala(L) |  | -23 | -3 | -17 | 5.42 | 1893 |
| Precuneus(R) | 7 | 6 | -51 | 63 | 4.45 | 79 |
| Precentral(R) |  | 47 | -20 | 63 | 4.32 | 213 |
| Postcentral Gyrus(R) |  | 53 | -14 | 54 | 4.04 | 65 |
| Middle Cingulum Gyrus(L) | 24 | -2 | -6 | 39 | 4.03 | 86 |
| Inferior Temporal Gyrus(L) |  | -48 | -72 | -2 | 3.86 | 99 |
| **Negative Connectivity** |  |  |  |  |  |  |
| Angular(R) |  | 52.5 | -54 | 39 | 5.31 | 724 |
| Middle Frontal Gyrus(L) |  | -42 | 12 | 45 | 5.00 | 2101 |
| Middle Temporal Gyrus(L) |  | -61.5 | -30 | -7.5 | 4.69 | 200 |
| Middle Frontal Gyrus(L) |  | -27 | 60 | 9 | 4.64 | 752 |
| Angular(L) | 40 | -49.5 | -55.5 | 46.5 | 4.64 | 595 |
| Frontal_Inf_Orb_R |  | 48 | 43.5 | -15 | 4.57 | 96 |
| Cuneus(L) | 31 | -13.5 | -55.5 | 27 | 4.17 | 161 |
| Frontal_Inf_Orb_L |  | -45 | 40.5 | -15 | 3.94 | 107 |
| Middle Frontal Gyrus(R) |  | 31.5 | 54 | -6 | 3.92 | 130 |
| Middle Frontal Gyrus(L) |  | -39 | 48 | -12 | 3.76 | 79 |

All clusters are significant at a threshold of p<0.001 and an extent threshold of p<0.05 with cluster-level family-wise error correction. Secondary local maxima within the significant clusters are not listed.
